# A Semantic and Knowledge-Based Approach for Handover Management

**DOI:** 10.3390/s21124234

**Published:** 2021-06-21

**Authors:** Fulvio Yesid Vivas, Oscar Mauricio Caicedo, Juan Carlos Nieves

**Affiliations:** 1 Departamento de Telemática, Universidad del Cauca, Popayán, Cauca 190002, Colombia; fyvivas@unicauca.edu.co; 2 Department of Computing Science, Umeå University, 901 87 Umeå, Sweden; juan.carlos.nieves@umu.se

**Keywords:** semantic information model, knowledge base profile, handover management, wireless networks

## Abstract

Handover Management (HM) is pivotal for providing service continuity, enormous reliability and extreme-low latency, and meeting sky-high data rates, in wireless communications. Current HM approaches based on a single criterion may lead to unnecessary and frequent handovers due to a partial network view that is constrained to information about link quality. In turn, HM approaches based on multicriteria may present a failure of handovers and wrong network selection, decreasing the throughput and increasing the packet loss in the network. This paper proposes SIM-Know, an approach for improving HM. SIM-Know improves HM by including a Semantic Information Model (SIM) that enables context-aware and multicriteria handover decisions. SIM-Know also introduces a SIM-based distributed Knowledge Base Profile (KBP) that provides local and global intelligence to make contextual and proactive handover decisions. We evaluated SIM-Know in an emulated wireless network. When the end-user device moves at low and moderate speeds, the results show that our approach outperforms the Signal Strong First (SSF, single criterion approach) and behaves similarly to the Analytic Hierarchy Process combined with the Technique for Order Preferences by Similarity to the Ideal Solution (AHP-TOPSIS, multicriteria approach) regarding the number of handovers and the number of throughput drops. SSF outperforms SIM-Know and AHP-TOPSIS regarding the handover latency metric because SSF runs a straightforward process for making handover decisions. At high speeds, SIM-Know outperforms SSF and AHP-TOPSIS regarding the number of handovers and the number of throughput drops and, further, improves the throughput, delay, jitter, and packet loss in the network. Considering the obtained results, we conclude that SIM-Know is a practical and attractive solution for cognitive HM.

## 1. Introduction

HM is responsible for making network (dis)connection decisions in a timely manner [1,2]. In this sense, HM is pivotal for providing service continuity, enormous reliability and extreme-low latency, and meeting sky-high data rates, in current and upcoming wireless communications [3,4]. To achieve efficient HM challenges need to be face that are related to high handover rates and ping-pongs in dense communication environments, leading to an increase in both the data flow latency and the packet loss and, consequently, to a reduction of the network throughput [5,6]. Users moving at moderate-to-high speed require a seamless handover mechanism with few failures [7,8].

In the networking literature, we find two types of approaches that address HM, namely, single criterion-based and multicriteria-based. Approaches based on a single criterion, such as the Signal Strong First (SSF), usually consider only the link quality in the end-user device for carrying out handovers. SSF compares the Received Signal Strength Indication (RSSI) of available networks and selects the network with the highest signal [9]. Single criterion-based approaches operate with a constrained network view that disregards contextual information, such as movement velocity and application requirements, leading to unnecessary and frequent handovers. These handover issues can decrease throughput, increase packet loss and even cause network service disruption [10,11,12]. The multicriteria-based approaches in [13,14,15,16,17,18,19,20,21,22] use RSSI and context information as criteria for ranking the available networks; the top-ranked network is selected by the end-user device for performing the connection process. These approaches disregard one or more relevant criteria, such as wireless network characteristics (e.g., coverage area), user device features (e.g., battery consumption), application requirements (e.g., real-time response) or user peculiarities (e.g., mobility pattern), leading to the failure of handovers and wrong network selection, negatively impacting the network’s performance [12,23]. Hybrid solutions combine multicriteria approaches [24]; however, their computational complexity is high.

This paper presents SIM-Know, an approach for improving HM. The contributions of SIM-Know are two-fold. SIM-Know proposes a Semantic Information Model (SIM) that allows us to make context-aware handover decisions by considering and relating criteria from several context information domains: Network, Application, User, UserDevice, and Handover. SIM-Know also introduces a SIM-based distributed Knowledge Base Profile (KBP) that offers local and global intelligence for making contextual and proactive decisions during the handover process. We evaluated SIM-Know in an emulated wireless network. When the end-user device moves at low and moderate speeds, the results show that our approach outperforms SSF (a single criterion approach) and behaves similarly to the Analytic Hierarchy Process combined with the Technique for Order Preferences by Similarity to the Ideal Solution (AHP-TOPSIS, a multicriteria approach), regarding the number of handovers and the number of throughput drops. SSF outperforms SIM-Know and AHP-TOPSIS regarding the handover latency metric because SSF runs a straightforward process for making handover decisions. At high speeds, SIM-Know outperforms SSF and AHP-TOPSIS regarding the number of handovers and the number of throughput drops and, further, improves the throughput, delay, jitter and packet loss in the network. From the obtained results, we conclude that SIM-Know is an attractive and feasible solution for cognitive HM.

The rest of this paper is organized as follows: Section 2 reviews the related work. Section 3 introduces SIM-Know, including SIM and KBP. Section 4 presents the evaluation of SIM-Know. Finally, some conclusions and future work are presented in Section 5.

## 2. Related Work

This section presents research on HM approaches based on both a single criterion and on multiple criteria. Table 1 briefly summarises each approach and presents the type of data/information model employed and how they make handover decisions. According to [25], the handover control can be: Network-Controlled HandOver (NCHO), Mobile-Controlled HandOver (MCHO), Mobile-Assisted HandOver (MAHO) and Network-Assisted HandOver (NAHO). In NCHO, the network starts and controls the handover; operators usually adopt it for load balancing and traffic management. In MCHO—traditionally used by IEEE 802.11 technologies—the end-user device initiates and controls the handover. In MAHO, the end-user device helps in the handover process controlled by the network; MAHO is typically used in cellular networks. In NAHO, the network helps with the handover process controlled by the end-user device; NAHO is intended for heterogeneous wireless networks.

Next, we point out the main shortcomings of the related work. The work in [9] employed SSF to make handover decisions. Since it does not exploit contextual information, such as movement velocity and application requirements for enhancing HM, SSF can lead to unnecessary and frequent handovers, decreasing throughput, increasing packet loss and even causing network service disruption. In [13,14,15,16,17,18,19,20,21,22] handover decisions are made by using multicriteria and techniques such as Fuzzy Logic, Network Management Policies or Multiple Attribute Decision Making (MADM) Algorithms. Nonetheless, these approaches neglect an information model that disregards criteria from one or more information domains, such as network characteristics and status, application requirements, end-user profile, end-user device features, or handover history, which are relevant for advancing HM. Thus, they can also lead to handover issues (generating failure, unnecessary and frequent handovers) and, consequently, network performance degradation. The work in [24] presents a solution that combines multicriteria approaches for improving HM. Nevertheless, its computational complexity is higher than that of the unique criterion and individual multicriteria methods. Unlike the related work, SIM-Know introduces an SIM to enable context-aware handover decisions by considering and relating criteria from a comprehensive set of context information domains: Network, Application, User, UserDevice and Handover. SIM-Know also proposes KBP, to realize cognitive and proactive handover decisions by providing local and global knowledge.

## 3. SIM-Know

HM allows an end-user device to keep an active connection when moving from one network’s coverage area (BS or AP coverage) to another [9]. HM comprises the initiation, selection and execution phases [17,26,27]. Handover Initiation gathers all the information needed to identify and determine the neighboring networks and their current and future statuses (e.g., data about network performance and available services). Network Selection chooses the best available network from a ranking created by taking into account a single criterion or multiple criteria. Handover Execution connects and disconnects end-users to and from a network, involving resource allocation and releasing [28].

SIM-Know introduces SIM and KBP for improving HM. SIM allows SIM-Know to make context-aware handover decisions. In turn, the distributed KBP provides local and global intelligence to make rule-based cognitive decisions about network connection and disconnection. SIM and KBP envision diminishing the number of handovers and the number of throughput drops and, as a consequence, have a positive impact on several network performance metrics (delay, jitter, packet loss, and throughput).

### 3.1. Semantic Information Model

**Figure 1:**
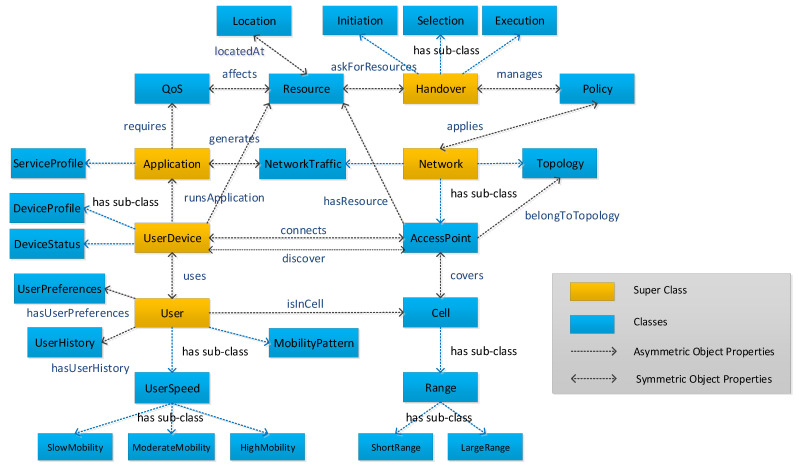
Semantic Information Model.

SIM-Know makes appropriate and contextual handover decisions by considering criteria from several information domains (i.e., *Network*, *Application*, *User*, *UserDevice*, and *Handover*) modeled by SIM. We use the CIM and the OWL to carry out SIM (see Figure 1). We adopted CIM [29] because it provides high expressiveness for modeling, for management purposes, information systems, applications, and networks [30]. We used OWL [31] because it enables reasoning in the model and the sharing of knowledge among software agents [32]. In particular, SIM uses OWL classes and properties to characterize HM entirely by modeling the information domains and their relationships.

Figure 1 shows the five knowledge domains comprising SIM as superclasses, namely, Network, User, Application, UserDevice and Handover. It is worth noting that the *Handover* superclass models HM by using the *Initiation*, *Selection*, *Execution* and *Policy* classes. The other superclasses represent the information domains containing the parameters used to improve decision making in HM. Each superclass relates to other classes by the *has sub-class* relationship. The *Initiation* class models the initiation phase that defines when the selection phase is triggered, which, in turn, is modeled by the *Selection* class that is responsible for obtaining the candidate networks for performing the handover. The *Execution* class performs the handover itself, since it allocates and releases AP (i.e., *AccessPoint* class) and user device (i.e., *UserDevice* class) resources, represented by the *Resource* class, which affects the QoS required (i.e., *QoS* class) by a user application (i.e., *Application* class). The *Policy* class represents the policies to apply to the *Network* class and governs the HM process. An example of a policy is to rank the candidate networks considering some criteria such as users’ speeds and their movement patterns.

The *Network* superclass models the characteristics and status of a network by using the *Topology*, *NetworkTraffic*, and *AccessPoint* classes. The *Topology* class represents the network’s organization, including nodes and links. The *NetworkTraffic* class represents data and control traffic passing by the network. The *AccessPoint* class models a networking device using wireless technology; this class considers the area covered by the *Cell* class, which includes the *Range* class, which contains the *LargeRange* and *SmallRange* classes. The *isCoveredByCell*, *hasResource* and *belongToTopology* properties represent the *AccessPoint* class’s relationship with the *Cell*, *Resource* and *Topology* classes, respectively. The *Resource* class models the ability to manage the resource consumption of APs located at (*Location* class) a particular network point.

The *User* superclass models the profile and behavior of the end-users by the *UserPreferences*, *UserHistory*, *MobilityPattern* and *UserSpeed* classes. The UserSpeed class includes the *SlowMobility*, *ModerateMobility* and *HighMobility* classes in order to model how fast an end-user moves. The *UserPreferences* class profiles the users with information related to, for instance, network preference by cost and service quality expectation. The *UserHistory* class models the historical (dis)connection of end-users. The *MobilityPattern* contains information about the end-users’ mobility pattern, which is predictable from their trajectory and velocity. The *Application* superclass represents end-user applications with the *ServiceProfile* and *QoS*. The *ServiceProfile* class models the application’s types (e.g., remote surgery, augmented reality, high definition video conferences). The *QoS* class allows the representation of a set of QoS requirements (e.g., delay, throughput and packet loss) for each type of application.

The *UserDevice* superclass models the end-user devices and their components by the *DeviceProfile*, *DeviceStatus* and *Resource* classes. The *DeviceProfile* class models the device’s characteristics. The *DeviceStatus* class represents the device’s current status (e.g., low-battery and off-air). The *Resource* class models the ability to manage the resource consumption of end-user devices located at (*Location* class) a particular network point. The *UserDevice* superclass relates to the *Application* superclass via the *runsApplication* property, which allows knowledge of the applications that are running in each end-user device. The *isUsedByUser* property defines a relationship between *UserDevice* and *User*.

### 3.2. Knowledge Base Profile

**Figure 2:**
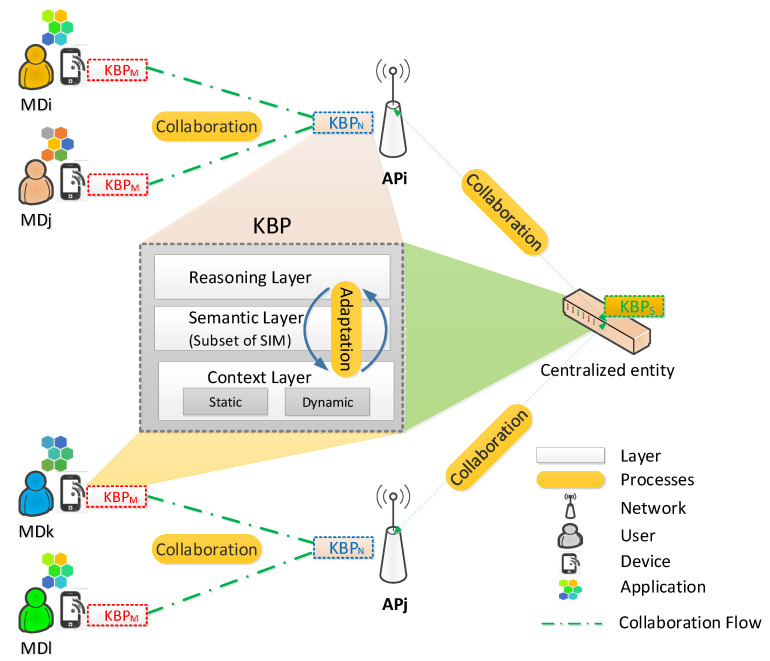
Knowledge Base Profile.

KBP is a distributed knowledge base that intends to provide local and global intelligence that supports making rule-based cognitive decisions about network connection and disconnection processes. Figure 2 depicts the KBP internal structure, which is comprised of layers and processes. The *Semantic* layer uses SIM (the entire model or a part) to obtain information from the data included in the *Context* layer. The *Reasoning* layer obtains knowledge from the information represented by SIM. The *Adaption* process acts on the layers to maintain updated data, information and knowledge. The *Collaboration* process enables the sharing of the obtained knowledge between KBP instances. Next, we detail the KBP’s layers and processes.

#### 3.2.1. Layers

The *Context* layer includes contextual data about the user, network, device, application, and handover. Contextual data are essential for carrying out HM in environments with multiple wireless networks [20]. As in [21,33], this layer is divided into static and dynamic sublayers. The *Static Context* sublayer involves data that do not change or rarely do; it plays a vital role in assisting with neighbor network discovery [34]. Examples of static data include the wireless technology supported by the end-user devices and APs, and the wireless network technology coverage area. The *Dynamic Context* sublayer serves an updated network view, including dynamic data such as the application requirements of a device needing handover and capacity available in a target AP, which enables the upper layers (*Semantic and Reasoning* layers) to realize knowledge-based handovers.

The *Semantic* layer offers a SIM instance that is nourished by the bottom layer’s contextual data. Thus, the *Semantic* layer structures the information to achieve intelligent, timely and context-aware HM (considering criteria from the Static and Dynamic contexts). For instance, the *DeviceProfile*, *UserPreferences*, *UserSpeed* and *MobilityPattern* SIM classes can be used to build up a map of candidate networks; overall, SIM classes provide a structure to contextual data. It is worth noting that we consider three KBP flavors depending on how they instantiate SIM. $KBP_{N}$, located at any AP or BS, instantiates the superclasses *Network* and *Handover*. $KBP_{M}$, located at end-user devices, instantiates the superclasses *Application*, *User*, *UserDevice* and *Handover*. $KBP_{S}$ is a complete KBP that can run on a logically centralised entity (e.g., a controller in a software-defined wireless network). The SIM’s distribution allows any KBP (SIM-Know) to create and share local knowledge to generate global knowledge.

The *Reasoning* layer triggers the Initiation phase, selects the target network and realizes the handover itself by inferring knowledge from SIM. We use Description Logic (DL) [35] to express in a structured and formal way the rules governing the *Reasoning* layer and, so, HM; the reasoning rules generate local and global intelligence to make autonomous handover connection decisions. Each rule has a set of conditions and settings. To illustrate how the *Reasoning* layer operates, next, we present some of the rules that are modeled to realize a policy intended to select candidate networks proactively, considering the coverage of APs and the mobility pattern of end-user devices. For example, the Rule APInRange (see Listing 1) serves to discover neighboring networks considering RSSI.

**Listing 1.** Rule for APInRange.
APInRange≡User⊓∃isInCell.(∃covers.AccessPoint)

Listing 2 shows that the Rule UserSpeed is useful for defining the speed of users. If a User is moving with $v>th_{u}$, he/she has a UserHighSpeed. If a User is moving with a speed higher than $th_{l}$ and lower than or equal to $th_{u}$, he/she has a UserModSpeed. UserSlowSpeed is when the user moves with $v\leq th_{l}$. According to [36], $th_{u}$ can be set to 50 Km/h and $th_{l}$ to 10 Km/h.

**Listing 2.** Rule for UserSpeed.
UserHighSpeed≡User⊓(∃hasUserSpeed.HighMobility)
UserModSpeed≡User⊓(∃hasUserSpeed.ModerateMobility)
UserSlowSpeed≡User⊓(∃hasUserSpeed.SlowMobility)

Listing 3 shows that Rule APRange is helpful for listing the APs by coverage range. LargeRange is given by $range>th_{r}$ and ShortRange by $range\leq th_{r}$. According to [14], $th_{r}$ can be set to 35 m for 802.11ac. Listing 4 presents Rule SoJournTime, which is useful for determining the time the user stays covered by an AP. If UserSlowSpeed is moving in APLRange, it results in LongSojournTime. If UserHighSpeed is moving in APSRange, it results in SmallSojournTime. A MSojournTime happens when UserHighSpeed or UserModSpeed is moving in APLRange. If UserSlowSpeed or UserModSpeed is moving in APSrange, it also results in MSojournTime. SmallSojournTime may be more challenging than LongSojournTime and MSojournTime in 5G networks and beyond is characterized by small coverage areas and high-mobility.

**Listing 3.** Rule for APRange.
APLRange≡AP⊓∃isCoveredBy.(∃hasRange.LargeRange)
APSRange≡AP⊓∃isCoveredBy.(∃hasRange.ShortRange)

**Listing 4.** Rule for SoJournTime.
LongSojournTime≡UserSlowSpeed⊓(∃APLRange)
SmallSojournTime≡UserHighSpeed⊓(∃APSRange)
MSojournTime≡APLRange⊓(UserHighSpeed⊔UserModSpeed)⊔
APSRange⊓(UserSlowSpeed⊔UserModSpeed)

Listing 5 shows that Rule CandidateAP is useful for creating the list of candidate APs for end-user devices with MSojournTime or LongSojournTime in the network. Listing 6 presents Rule AssociateAP, which links the end-user device with the first AP in the list of candidates. It is worth noting that each network administrator can define his/her own rules to manage the wireless network as he/she needs.

**Listing 5.** Rule for CandidateAP.
CandidateAP≡MSojournTime⊔LongSojournTime

**Listing 6.** Rule for AssociateAP.
AssociationToAP≡User⊓∃Uses.UserDevice(∃Connects.AP)

#### 3.2.2. Processes

The *Adaptation* process allows SIM-Know to dynamically adapt to the changing environments and to enhance HM by modifying the content of the layers of the KBP instances. The content is modified in a bottom-up way, starting with the contextual data, followed by the SIM instances, and ending with the acquired knowledge when environmental changes happen, such as new networks appearing, dynamic traffic conditions and variations in QoS requirements. Furthermore, this process allows the addition and updating of the *Reasoning* layer seeking to meet QoS and to preserve network performance.

The *Collaboration* process allows KBP ($KBP_{N}$, $KBP_{S}$, $KBP_{M}$ and any other profile defined to extend SIM-Know) to interchange the knowledge obtained for enhancing the decision-making in HM. For instance, the collaboration between $KBP_{M}$ and $KBP_{N}$ would allow for choosing the optimal and appropriate time to trigger the handover and select the most suitable access network according to the end-user QoS requirements and network status.

### 3.3. Operation

**Figure 3:**
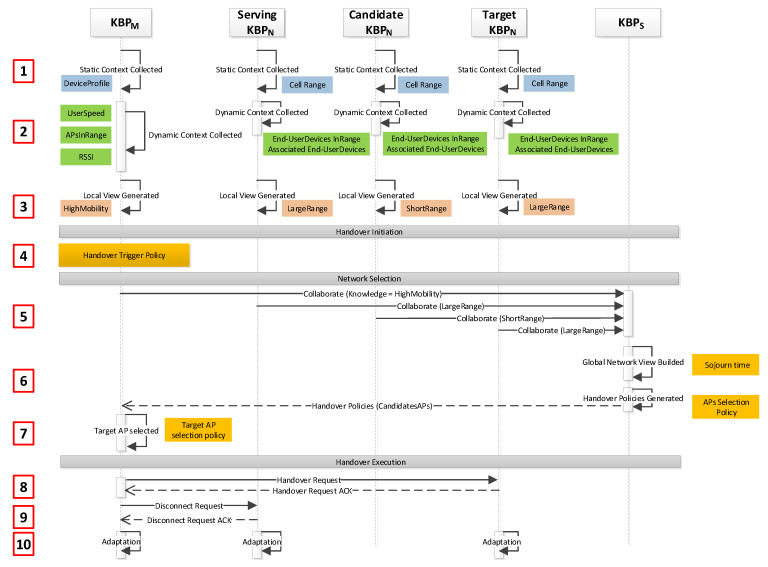
SIM-Know Operation.

Figure 3 presents how SIM-Know operates in WLAN. First, $KBP_{S}$, $KBP_{M}$ and $KBP_{N}$ collect their *Static Context*. Second, $KBP_{M}$ monitors and updates the *Dynamic Context* information related to, for instance, end-user speed, APs in range and RSSI. In parallel, $KBP_{M}$ requests from $KBP_{N}$ the *Dynamic Context* information, which includes the associated and in range end-user devices. Third, based on the *Static Context* and *Dynamic Context*, every KBP generates its local knowledge. For example, the local knowledge in $KBP_{M}$ can be HighMobility and in $KBP_{N}$ can be LargeRange. Fourth, $KBP_{M}$ launches the handover process. Fifth, the *Collaboration* process starts between the corresponding $KBP_{M}$ and $KBP_{N}$ and ends with sending knowledge to $KBP_{S}$. Sixth, $KBP_{S}$ builds up the global network view, generates a handover policy for selecting candidate APs according to the rules defined in its *Reasoning Layer*, and sends those candidates to $KBP_{M}$. Seventh, $KBP_{M}$ selects the *Target*$KBP_{N}$ by using the rules defined in its *Reasoning Layer*. Eighth, $KBP_{M}$ sends a Handover Request to the *Target*$KBP_{N}$, which sends back an acknowledgment to $KBP_{M}$. Ninth, $KBP_{M}$ sends disconnection requests to the current *Serving*$KBP_{N}$, which, in turn, sends a disconnection acknowledgment to $KBP_{M}$. Tenth, every KBP executes the *Adaptation* process to handle the context variations dynamically.

**Figure 4:**
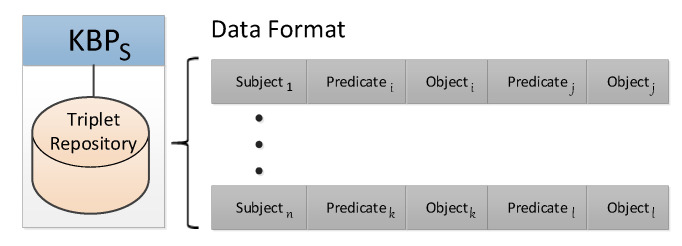
$KBP_{S}$ Data Format.

Figure 4 shows the data format used in $KBP_{S}$ to store the global knowledge built with the information coming from $KBP_{N}$ and $KBP_{M}$. The format follows the triplet (Subject, Predicate, Object) encoded in Entity Notation [37], which enables a lightweight knowledge representation for resource-constrained environments. The Subject identifies a class in SIM by the combination of ClassId (e.g., ceu101) and ClassType (e.g., *User*). Each Subject is related to various pairs, Predicate–Object. The Predicate identifies a property (e.g., UserSpeed) of the Subject while the Object provides the value of such a property (e.g., HighMobility). The Object can also be a ClassId, to represent relationships among Subjects.

**Figure 5:**
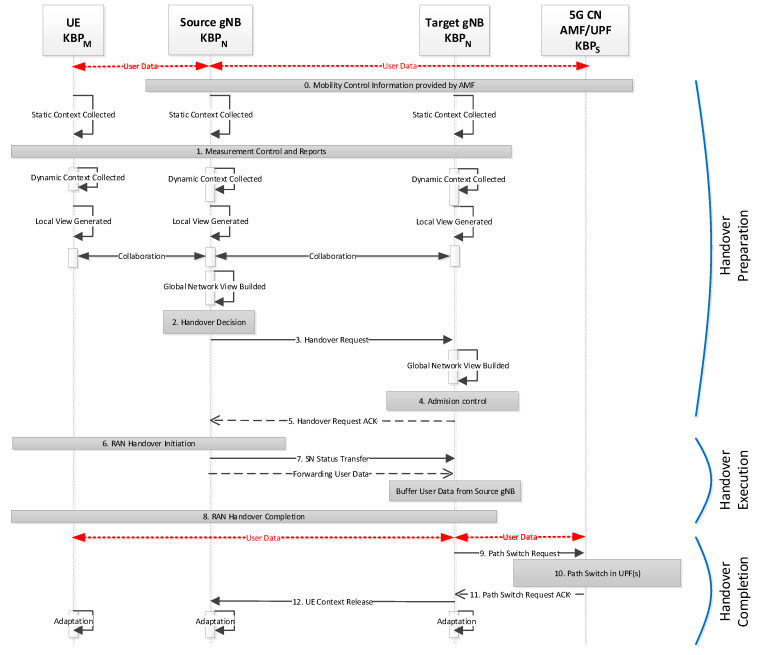
SIM-Know in 5G Intra-AMF/UPF Handover.

Figure 5 illustrates how SIM-Know can operate in 5G by running $KBP_{M}$ in the User Equipment (UE), $KBP_{N}$ in gNodeB (gNB), and $KBP_{S}$ in the Core Network (CN). The handover in 5G, according to the specification 3GPP TS38.300 [38], consists of three phases: preparation (steps 0–5), execution (steps 6–8), and completion (steps 9–12).

The steps are as follows:

- Step 0: Each KBP gathers the *Static* context.
- Step 1: $KBP_{M}$ and $KBP_{N}$ initialize measuring procedures to collect the *Dynamic* context, generate local knowledge, and exchange Measurement Reports by way of the *Collaboration* process. Source $KBP_{N}$ builds up its local knowledge.
- Step 2: Source $KBP_{N}$, based on its local knowledge, makes handover decisions. gNBs are responsible for making handover decisions.
- Step 3: Source $KBP_{N}$ sends a handover request message to Target $KBP_{N}$.
- Step 4: Target $KBP_{N}$ executes the admission control procedure based on its local knowledge.
- Step 5: Target $KBP_{N}$ sends a handover request acknowledgment to Source $KBP_{N}$.
- Step 6: Source $KBP_{N}$ sends a handover command to $KBP_{M}$ for handover initiation.
- Step 7: Source $KBP_{N}$ sends the sequence number status transfer message to Target $KBP_{N}$. Source $KBP_{N}$ may initiate data forwarding.
- Step 8: $KBP_{M}$ detaches from Source $KBP_{N}$ and synchronizes with Target $KBP_{N}$.
- Step 9: Target $KBP_{N}$ informs $KBP_{S}$ that $KBP_{M}$ has changed the cell by way of the path switch request message.
- Step 10: $KBP_{S}$ switches the data path towards Target $KBP_{N}$.
- Step 11: $KBP_{S}$ acknowledges the path switch request message.
- Step 12: Target $KBP_{N}$ informs Source $KBP_{N}$ that the handover was successful and triggers the release of resources for Source $KBP_{N}$ by sending a UE Context Release message. Finally, Source $KBP_{N}$ releases the resources associated with $KBP_{M}$, invoking the *Adaptation* process.

## 4. Evaluation

This section presents the evaluation of SIM-Know in a WLAN, aiming to show its behavior regarding the number of handovers and the number of throughput drops, and its impact on various typical network performance metrics. Section 4.1 depicts the SIM-Know’s prototype and the test environment. Section 4.2 shows the performance metrics and traffic generation. Section 4.3 presents and discusses the results of SIM-Know and two well-known handover solutions.

### 4.1. Prototype and Test Environment

**Figure 6:**
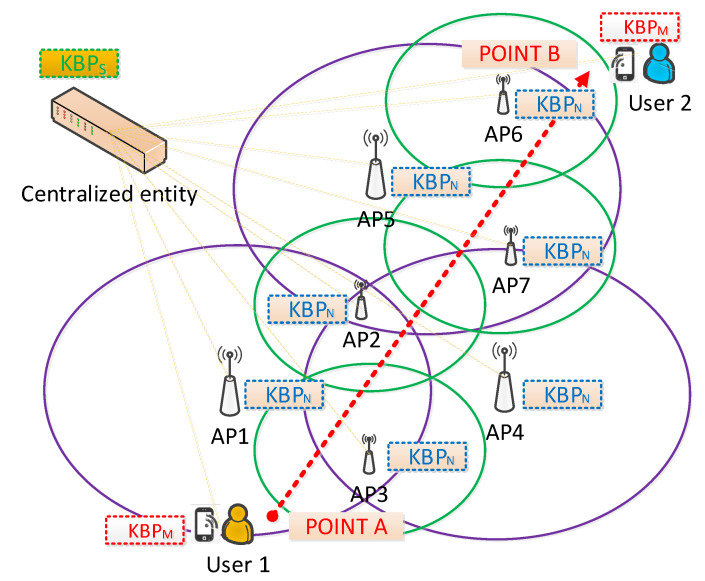
Test Environment.

We implemented the SIM-Know prototype for WLAN, including $KBP_{M}$, $KBP_{N}$, and $KBP_{S}$, by using the Python programming language version 2.7. We also deployed the prototype in a Mininet-WiFi emulator [39] (see Figure 6) running on an Ubuntu 16.04 VM with a Core i7-3630 processor and 8 GB RAM. Mininet-WiFi adds virtual BSs and APs to classical Mininet [40] to enable the emulation of wireless network environments. The SIM-Know prototype, as well as all test scripts, are available in the project repository [41].

Figure 6 shows the WLAN test scenario in which we evaluated and compared SIM-Know, SSF, and AHP-TOPSIS. The scenario, deployed in Mininet-WiFi, included seven APs, an end-user device associated with User1, and another end-user device linked to User2. In particular, we used three APs with a large coverage range (i.e., up to 75m for $AP_{1}$, $AP_{4}$, and $AP_{5}$ with 802.11n) and four with a short coverage range (i.e., up to 35m for $AP_{2}$, $AP_{3}$, $AP_{6}$, and $AP_{7}$ with 802.11ac). We also analyzed the performance when the User 1 moved from Point A to Point B by following a straight line without directional change at a constant speed. We used three speeds for testing: 1.42 m/s corresponding to SlowMobility, 3.74 m/s to ModerateMobility, and 13.41 m/s to HighMobility. The end-user device associated with User 1 transmitted traffic (Voice over IP (VoIP) or Transmission Control Protocol (TCP)) to the end-user device linked to User 2, which was immobile. We repeated the experiments thirty-three times to obtain results with a 95% confidence level. Table 2 summarizes the setup of the experiments.

It is worth mentioning that the described scenario was constrained to a small number of end-user devices because our main objective was to show the feasibility of SIM-Know. We will perform more extensive evaluations on large emulated environments in our subsequent papers.

### 4.2. Performance Metrics and Traffic Generation

We compared SIM-Know to SSF and AHP-TOPSIS in terms of the number of handovers, number of throughput drops, handover latency, and various well-known network performance metrics (throughput, delay, jitter and packet loss) [42]. The quantity of handovers is the number of transfers an end-device makes when it moves from one place to another [43]. The throughput drops represent the times that the number of bytes transmitted falls to zero because of a handover [28]. The handover latency is the time that elapses between the instant that the user-device sends the last link-going-down message to the serving AP and the moment at which the end-user device establishes the connection with the target AP [3].

In the emulation experiments, scripts for generating traffic were developed by using the iPerf3 [44] and D-ITG [45] tools. We used D-ITG to generate VoIP flows with audio code (G.711.2-84 Kbps and 50 pkts/s) transmitted using real-time protocol and voice activity detection. We used iperf3 to generate TCP flows with constant inter-departure time between packets (1000 pkts/s) and constant packet size (512 bytes).

### 4.3. Results and Analysis

Table 3 shows that SIM-Know and AHP-TOPSIS outperformed SSF, in terms of the number of handovers and the number of throughput drops, when the end-user device moved at any speed (slow, moderate, or high). This behavior is expected because SSF is the baseline and triggers a handover as soon as an AP with an RSSI higher than that of the serving AP is available.

**Figure 7:**
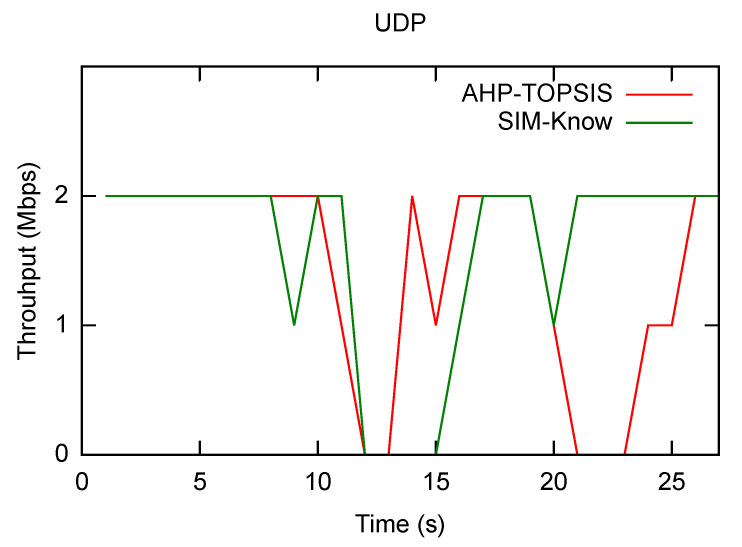
Throughput Drops.

**Figure 8:**
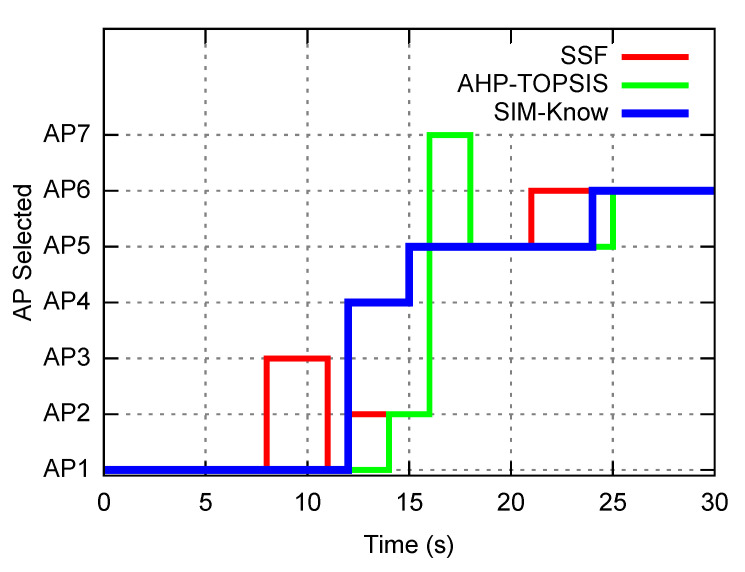
Proactivity.

Table 3 also reveals that when the end-user device moved at slow and moderate speeds, SIM-Know behaved as AHP-TOPSIS does regarding the number of handovers and the number of throughput drops. Figure 7 shows that SIM-Know outperformed AHP-TOPSIS in these metrics when the end-user device moved at a high velocity. The outperformance regarding the number of handovers and number of throughput drops was due to SIM-Know making context-aware, cognitive and proactive handovers. Figure 8 corroborates the fact that SIM-Know carried out handovers before SSF and AHP-TOPSIS did.

**Figure 9:**
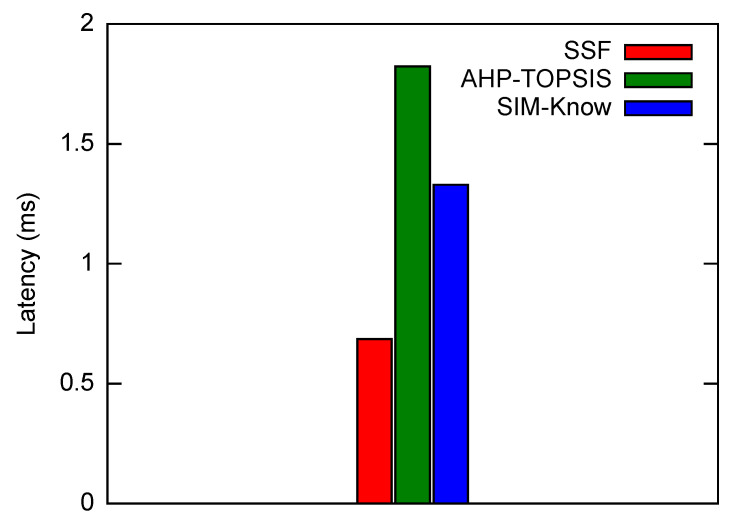
Handover Latency.

Figure 9 shows, as expected, that SIM-Know obtained a higher handover latency than SSF did, since our approach is knowledge-based and SSF makes decisions considering a single criterion. SIM-Know had 27% less handover latency than AHP-TOPSIS because, first, our approach is proactive and, according to [46], AHP-TOPSIS is reactive; the proactivity shortens the Handover Initiation phase [21]. Secondly, SIM-Know employs a rule-based reasoning method while AHP-TOPSIS uses a complex mathematical model that requires a considerable amount of time to make handover decisions.

**Figure 10:**
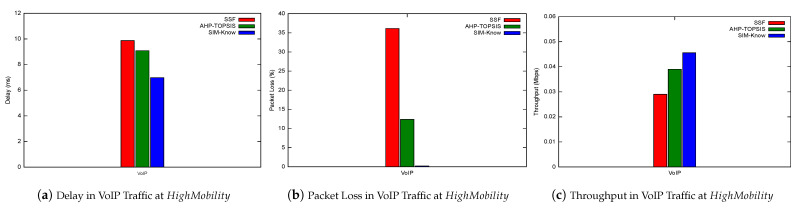
Impact on VoIP Traffic.

**Figure 11:**
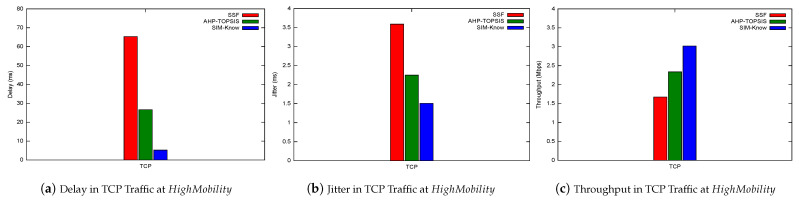
Impact on TCP Traffic.

Next, we present how SIM-Know, SSF and AHP-TOPSIS impact various network performance metrics when the end-user device moves at HighMobility. Figure 10 depicts SIM-Know overcoming SSF and AHP-TOPSIS regarding the throughput, delay, and packet loss when the wireless network transferred VoIP/UDP traffic. In particular, the delay attained by SIM-Know was 29.28% and 23.13% lower than that achieved by SSF and AHP-TOPSIS (see Figure 10a). The packet loss of SIM-Know was 99.44% and 98.38% lower than that obtained by SSF and AHP-TOPSIS (see Figure 10b). The throughput obtained by SIM-Know was 57.17% and 16.87% higher than that obtained by SSF and AHP-TOPSIS (see Figure 10c). Figure 11 shows that SIM-Know also outperformed SSF and AHP-TOPSIS regarding the throughput, delay and jitter when the wireless network transferred TCP traffic. Specifically, the delay attained by SIM-Know was 91.95% and 80.27% lower than that achieved by SSF and AHP-TOPSIS (see Figure 11a). The jitter performed by SIM-Know was 57.98% and 32.94% lower than that obtained by SSF and AHP-TOPSIS (see Figure 11b). The throughput accomplished by SIM-Know was 80.3% and 29.22% higher than that attained by SSF and AHP-TOPSIS (see Figure 11c).

We argue that the improvement in throughput, delay, jitter, and packet loss offered by SIM-Know is due to its context-aware, cognition and proactivity capabilities, which decreased the number of handovers and the number of throughput drops. In particular, SIM provides the context-aware capability to perform handover decisions through the comprehensive and semantic network view given by the information domains (*Network*, *Application*, *User*, *UserDevice*, and *Handover*). The KBP’s *Reasoning* layer allows the achievement of cognitive HM. The distributed nature of KBP and its continuous updating allow the Handover Initiation phase to be proactive and operate with the local knowledge, built by $KBP_{M}$ and $KBP_{N}$, and the global knowledge, available in $KBP_{S}$. It is worth noting that the above results corroborate the idea that proactive approaches are more effective than reactive ones for meeting QoS requirements. On the other hand, we consider that the handover latency of SIM-Know can be addressed by improving the data format and the communication model between $KBP_{S}$ and both $KBP_{M}$ and $KBP_{N}$. These improvements are out of the scope of this paper and constitute limitations of the current SIM-Know version.

## 5. Conclusions and Future Work

This paper introduced SIM-Know, an approach that performs context-aware, cognitive, and proactive handovers. SIM-Know includes SIM to provide context-awareness to handover decisions and KBP to incorporate cognition in HM. KBP distributes knowledge (local and global) to afford the proactivity capability in HM. The evaluation results showed that, thanks to the aforementioned SIM-Know capabilities, our approach overcomes SSF regarding the number of handovers and the number of throughput drops when the end-user device moves at any speed and, further, equals AHP-TOPSIS when it travels at low and moderate speeds. SSF outperforms SIM-Know and AHP-TOPSIS regarding the handover latency metric because SSF runs a straightforward process for making handover decisions. SIM-Know overcomes AHP-TOPSIS regarding all evaluated metrics when the end-user device moves at a high speed, positively impacting the wireless network’s performance in terms of the delay, throughput, packet loss and jitter metrics. Considering these results, we concluded that SIM-Know is an attractive and feasible solution for cognitive HM.

For future work, we intend to enrich our approach with SDN and NFV capabilities to deal with the scalability issue imposed on HM by the Industrial IoT and the massive IoT 5G use case. We also plan to create an efficient model for communicating KBPs and evaluating SIM-Know when it makes handover decisions in scenarios with many end-user devices, high network traffic, and high load in APs.

## Figures and Tables

**Table 1:** Related Work.

Work	Description	Making-Decision		Wireless Technology
		Control	Method	
[9]	The end-user device switches to another AP or BS when the RSSI level of the serving network is lower than a threshold	MCHO	Policy	Universal Mobile Telecommunications System, Wireless Local Area Network (WLAN)
[13]	A Software-Defined Networking (SDN) controller uses a fuzzy system to score candidate networks for staying in the current network or connecting to a better one	NCHO	Fuzzy Logic	Fifth Generation (5G)
[14]	An algorithm is proposed to reduce the handovers by multicriteria decision-making algorithms improved with a context-aware and threshold-based scheme	MCHO	TOPSIS, PROMETHEE, SAW	5G, Long-Term Evolution (LTE), WLAN
[15]	A framework, based on a quantum-inspired immune clonal algorithm and network-related and user-related parameters with different weights, is proposed for improving the access network selection in wireless networks	NCHO	Quantum Computing	LTE, WLAN
[16]	A fuzzy logic and reinforcement learning-based mechanism is introduced to address unnecessary and frequent handovers by adjusting the handover hysteresis margin and time-to-trigger	NCHO	Fuzzy Q-Learning	LTE
[17]	A solution, based on SDN, Binary Integer Linear Programming (BILP), user criteria and network packet error rate data, is proposed to rank candidate BS and to enhance the handover selection phase	NCHO	BILP	LTE
[18]	A framework, based on data analytics, context extraction, user profiling and pre-processing contextual information, is presented to score the available BS and to improve network access selection	NAHO	Fuzzy Logic	5G
[19]	The AP or BS selection is improved by using AHP for weighting selection criteria coming from the user and networks’ context and TOPSIS for ranking the available networks	NCHO	AHP-TOPSIS	LTE, WLAN
[20]	A mechanism is proposed for selecting the radio access network that best meets the end-user needs by considering the on/off state and battery level of the mobile device and the available bandwidth in the target and serving network	MCHO	Policy	LTE, WLAN
[21]	A versatile modeling methodology is introduced for evaluating proactive and reactive vertical handover approaches	NCHO	Policy	5G, LTE, WLAN
[22]	Two co-operating algorithms with adaptive thresholds are introduced for performing network selection while avoiding network congestion and meeting user preferences regarding monetary cost, QoS, security and energy consumption	NAHO	Policy	5G
[24]	A multiattribute decision handover making scheme, centered in the triggering phase and based on SDN and Fuzzy Logic, is proposed for increasing the network throughput and reducing unnecessary handovers and total handover delay in femto-access points and device-to-device communications	NAHO	TOPSIS, Fuzzy Logic, AHP	LTE, WLAN

PROMETHEE: Preference ranking organization method for enrichment evaluation—SAW: Simple Additive Weighting Algorithm.

**Table 2:** Experiment Setup.

Parameters	Value
Wireless technology	802.11n, 802.11ac
Emulation area	180 × 180 m
Carrier frequency	2 GHz
Channel bandwidth	20 MHz
Transmission power of cells large-range/short-range	25/14 dBm
Path loss model from cells	Log-Distance Propagation Loss/ITU-R P1283
Emulation time for HighMobility	30 s
Emulation time for ModerateMobility	80 s
Emulation time for SlowMobility	180 s
TCP traffic	Flows with constant inter-departure time between packets (1000 pkts/s) and constant packets size (512 bytes)
VoIP traffic	Flows with audio code (G.711.2 - 84 Kbps and 50 pkts/s) transmitted using real time protocol and voice activity detection

**Table 3:** Handover Performance.

Parameter	SSF	AHP-TOPSIS	SIM-Know
**SlowMobility**			
Number of handovers	7	3	3
Number of throughput drops	5	3	3
**ModerateMobility**			
Number of handovers	7	3	3
Number of throughput drops	5	3	3
**HighMobility**			
Number of handovers	7	4	3
Number of throughput drops	5	2	1

## Data Availability

The SIM-Know Project Repository. 2021. Available online: https://github.com/fyvivas/SIMKnow (accessed on 11 June 2021).

## Abbreviations

The following abbreviations are used in this manuscript:

3GPP	3rd Generation Partnership Project
5G	Fifth Generation
AHP	Analytic Hierarchy Process
AMF	Access and Mobility Management Function
AP	Access Point
BILP	Binary Integer Linear Programming
BS	Base Station
CIM	Common Information Model
CN	Core Network
DL	Description Logic
EN	Entity Notation
gNB	gNodeB
HF	Handover Failure
HM	Handover Management
IoT	Internet of things
KBP	Knowledge Base Profile
LTE	Long-Term Evolution
MADM	Multiple Attribute Decision Making
MAHO	Mobile-Assisted Handover
MCHO	Mobile-Controlled Handover
NAHO	Network-Assisted Handover
NCHO	Network-Controlled Handover
NFV	Network Function Virtualization
OWL	Web Ontology Language
PP	Ping-Pong
PROMETHEE	Preference Ranking Organization METHod for Enrichment Evaluation
QoS	Quality of Service
RAN	Radio Access Network
RLF	Radio Link Failure
RSSI	Received Signal Strength Indication
SAW	Simple Additive Weighting
SDN	Software-Defined Networking
SIM	Semantic Information Model
SSF	Signal Strong First
TCP	Transmission Control Protocol
TOPSIS	Technique for Order Preferences by Similarity to the Ideal Solution
UDP	User Datagram Protocol
UE	User Equipment
UPF	User Plane Function
URLLC	Ultra-Reliable and Low Latency Communications
VM	Virtual Machine
VoIP	Voice over IP
WLAN	Wireless Local Area Network
